# Oral versus intravenous sildenafil for pulmonary hypertension in neonates: a randomized trial

**DOI:** 10.1186/s12887-022-03366-3

**Published:** 2022-05-27

**Authors:** Chinmay Chetan, Pradeep Suryawanshi, Suprabha Patnaik, Naharmal B. Soni, Chandra Rath, Prince Pareek, Bhvya Gupta, Reema Garegrat, Arjun Verma, Yogen Singh

**Affiliations:** 1 Department of Neonatology, Himalayan Institute of Medical Sciences, Dehradun, Uttarakhand - 248140 India; 2 Department of Neonatology, Bharati Vidyapeeth University Medical College, Pune-Satara road, Pune, Maharashtra- 411043 India; 3 Department of Neonatology, Sidra Medicine, 26999 Doha, Qatar; 4 Department of Neonatology, Perth Children’s Hospital, 15 Hospital Ave, Nedlands, WA 6009 Australia; 5 Sparsh Superspeciality Hospital, Ambala, Haryana 134003 India; 6 Division of Neonatology, Loma Linda University School of Medicine, 11175 Anderson Street Rm 11121, Coleman Avenue, Loma Linda, 92354 USA

**Keywords:** Intravenous sildenafil, Neonates, Oral sildenafil, Pulmonary Hypertension

## Abstract

**Background:**

Sildenafil is the drug of choice for neonatal pulmonary hypertension in developing countries where inhaled nitric oxide is not available. Available as oral and intravenous preparation – no study has been done in the past to compare the two forms. Each has its own benefits – but requires comparison in terms of efficacy and safety. This study was done to compare the efficacy of oral versus intravenous (IV) sildenafil in infants with mild to moderate pulmonary hypertension.

**Methods:**

An open labelled randomized trial was conducted in a neonatal intensive care unit of urban tertiary hospital in western India between February 2019 to December 2020. Infants born after 34 weeks of gestation with Pulmonary arterial pressure (PAP) > 25 mm Hg measured by echocardiography, within 72 h of birth, were enrolled for the study. Participants were randomly assigned to receive sildenafil either orally or by intravenous route. Primary outcome was the time taken for PAP to decrease below 25 mm Hg. Secondary outcomes were time taken for oxygenation index to decrease by 25%, duration of invasive and non-invasive mechanical ventilation, nasal oxygen, hospital stay, time to achieve full feeds, mortality, and side effects.

**Results:**

Forty patients were enrolled. The baseline characteristics of neonates in both groups were similar except for APGAR scores at 1 min and 5 min, with oral group having lower score [MEDIAN (IQR) 5.00 (4.00- 7.00) and 7.00 (6.00- 8.00)] compared to IV group [MEDIAN (IQR) 7.00 (6.00–8.00) and 9.00 (8.00–9.00)] respectively. Time taken for PAP to decrease below 25 mm was not statistically different between the oral and intravenous groups. Systemic hypotension occurred in 4 neonates of the intravenous group but none in the oral group.

**Conclusion:**

Oral and intravenous sildenafil had equal efficacy at reducing PAP in neonatal pulmonary hypertension, albeit intravenous sildenafil use was associated with a greater complication rate.

**Trial registration:**

Trial was registered in the clinical trials registry of India [CTRI/2019/04/018781][25/04/2019].

**Supplementary Information:**

The online version contains supplementary material available at 10.1186/s12887-022-03366-3.

## Background

Despite new advances in management of persistent pulmonary hypertension of newborn (PPHN), mortality continues to be high, ranging from 4 to 33% [1, 2]. The standard treatment of PPHN in high-income countries, with inhaled nitric oxide (iNO) and extracorporeal membrane oxygenation (ECMO), is expensive and is not easily available in most resource-limited settings [2]. Pulmonary vasodilators, like Sildenafil, Magnesium sulfate, Milrinone, and Bosentan etc. are the mainstay of treatment in resource poor setting. Many trials have been undertaken recently to evaluate the efficacy of oral and intravenous (IV) Sildenafil in PPHN. Though most of these studies are underpowered, they suggest a beneficial role of this drug in neonates with PPHN [3–6]. A 2017 Cochrane review concluded that ‘Sildenafil used for treatment of pulmonary hypertension has potential for reducing mortality and improving oxygenation in neonates, especially in resource-limited settings where iNO is not available [7].’ While Sildenafil has been compared against other vasodilators or placebo [8–10], we could not find any trial comparing the efficacy and side effects of orally versus IV administered Sildenafil in PPHN. Oral Sildenafil although cheaper with easier route of administration, needs to be compared against IV Sildenafil and it is pertinent to look at the efficacy and side effects of these two forms of Sildenafil in a randomized control trial (RCT) setting beside exploring the pharmacokinetics, bioavailability and financial cost. In absence of any RCT this pilot study was planned.

## Materials and Methods

An open labelled, parallel, randomized trial was designed and conducted in a level III neonatal intensive care unit (NICU) in an urban academic medical centre in Pune, India. The study was approved by an institutional ethics committee and registered in the clinical trials registry of India [[CTRI/2019/04/018781][25/04/2019]. Enrolment period was from February 2019 to December 2020.

Late preterm and term infants with Pulmonary arterial pressure (PAP) > 25 mm Hg on echocardiography within 72 h of birth were enrolled [11, 12]. Neonates with a congenital heart disease (except patent ductus arteriosus, patent foramen ovale, atrial septal defect, or a single muscular ventricular septal defect of < 4 mm size), congenital diaphragmatic hernia, any lethal congenital anomaly, or with any contraindication for oral or IV sildenafil (systemic hypotension, necrotizing enterocolitis [NEC], or gastrointestinal bleeding), were excluded. Neonates who were treated with iNO (Oxygenation index (OI) > 15 or at the physician’s discretion) as first line treatment were also excluded, as it would have been unethical to deprive babies with severe pulmonary hypertension of iNO, which has been established as first line drug for pulmonary hypertension. We aimed to enroll a sample size of 40 patients based on likely enrollment rates in our nursery over the 2-year period as a pilot trial. All late preterm and term infants admitted in NICU, on nasal oxygen, non-invasive respiratory support or invasive respiratory support were screened for pulmonary hypertension by echocardiography every 24 h during the first 72 h after birth. Echocardiography was done on a Siemenes Acuson X 300 machine using a neonatal probe (4–8 Hz transducer). Pulmonary pressures were measured by tricuspid regurgitation velocity.

All consecutive neonates meeting the inclusion criteria were enrolled. After taking written informed consent from parents for study participation and publication of identification data, neonates were randomly assigned using computer generated numbers to either the oral or the IV sildenafil group in 1:1 ratio. PAP, OI, mechanical ventilation settings, and feeding status at the start of study were noted. Babies in the oral sildenafil group were started on 6-hourly oral sildenafil at 1 mg/kg/dose. For this, a 20 mg tablet of sildenafil (Penegra, Zydus Healthcare) was dissolved in 20 ml distilled water and the calculated dose was fed through an orogastric tube. Babies in the IV group were given a loading dose of 0.4 mg/kg of sildenafil over 3 h followed by continuous infusion of 1.6 mg/kg/day. For this, IV sildenafil (10 mg in 12.5 ml; Pulmosil, Sun Pharamaceuticals) was diluted in saline to the required concentration and infused at 1 ml/hr.

After starting Sildenafil, functional echocardiography was repeated every 12 h until PAP dropped to < 25 mmHg. Sildenafil was tapered when PAP reached < 25 mmHg and stopped within 4 days according to the hospital protocol. During tapering, echocardiography was done every 24 h. In case of rebound increase in PAP to > 25 mm Hg, Sildenafil was increased back to its original dose. Primary outcome was time taken for pulmonary pressures to reduce to below 25 mm Hg. Secondary outcomes were time for OI to decrease by 25%, days on invasive ventilation, non-invasive ventilation & nasal oxygen, duration of hospital stay, outcome, failure of treatment, complications and time to reach full feeds. During sildenafil treatment, all complications which could be attributed to the drug like hemodynamic instability, bleeding from any site, retinopathy of immaturity or any other adverse effect were monitored for. Arterial blood gas measurements were done as per unit policy. Hypotension was defined as blood pressure less than 10th centile according to gestation and postnatal age [13]. Echocardiography was done by a pre-designated person (treating clinician) who had received training in functional 2D echocardiography. To assess interobserver and intra-observer agreements, echocardiography in 20 random neonates, were repeated by the same observer and also by another trained clinician. Because of the study design, treating clinician could not be blinded as he also performed the echocardiography.

Data was analysed with SPSS statistical software (version 25). Continuous variables were summarized with standard descriptive statistics including means, standard deviations, medians and ranges. Categorical variables were summarized using means, frequencies and percentage. An independent sample t test was used to compare two independent numeric groups. Chi square test was used to find an association between two categorical variables. Fisher's exact test was used to find out the association between two categorical variables when observed frequency was less than five in at least one cell. Interclass correlation coefficients (ICC) was used to assess interobserver variability.

## Results

**Fig. 1:**
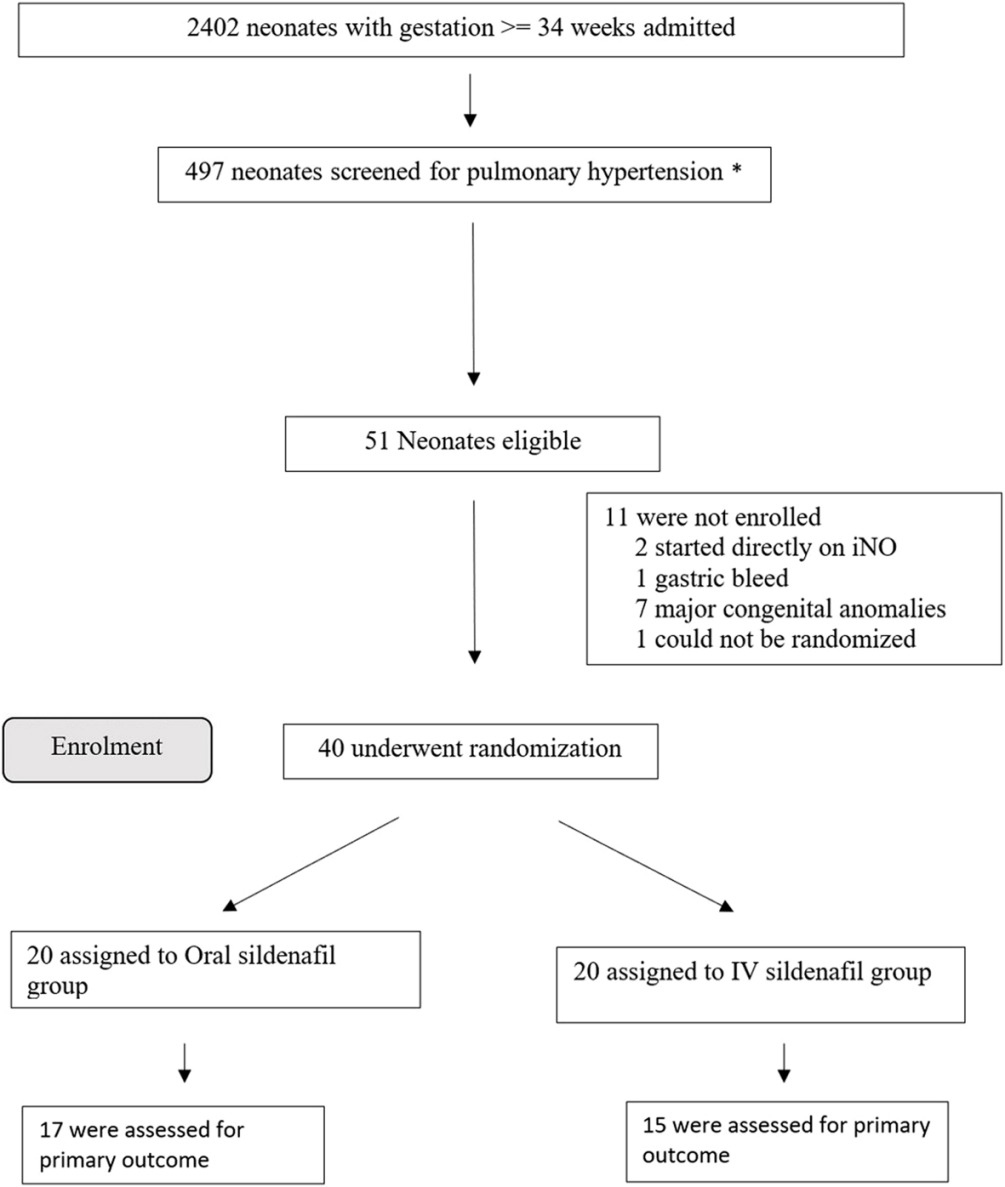
Study flow diagram. *497 neonates were on nasal oxygen, non-invasive respiratory support or invasive respiratory support

During the study period, 2,402 neonates of ≥ 34 weeks of gestation were admitted in the NICU. Fifty-one of these neonates were diagnosed to have mild to moderate PPHN. Eleven patients met exclusion criteria and the remaining 40 were included in the trial and randomized into the oral or IV sildenafil groups (Fig. 1). Baseline characteristic of the population is depicted in Table 1. Median gestation age and birth weight of the included neonates was 38.55 (IQR 36.25—40) weeks and 2792.5 (IQR 2376.25 – 3196.25) grams respectively. Neonates in the oral and IV groups were similar except for APGAR scores at 1 min and 5 min, with oral group worse at both time intervals [MEDIAN(IQR) 5.00 (4.00- 7.00) and 7.00 (6.00- 8.00)] for oral group and [MEDIAN(IQR) 7.00 (6.00–8.00) and 9.00 (8.00–9.00)], for IV group. Resuscitation was required more in the oral group (13/20) compared to the IV group (8/20), but was not statistically significant (*p* > 0.05). Meconium aspiration syndrome was present in 50% cases in oral group and 30% cases in IV sildenafil group (*p* = 0.102).

**Table 1:** Baseline characteristics of neonates with PPHN treated with oral or IV sildenafil

	**Oral sildenafil** ***n* = 20**	**IV sildenafil** ***n* = 20**	***P* value**
**Maternal characteristics**			
Primigravida	10 (50%)	9 (45%)	0.752
**Medical condition**			
Hypothyroidism	3 (15%)	4 (20%)	> 0.99
Diabetes	3 (15%)	2 (10%)	> 0.99
Hypertensive disorder	2 (10%)	3 (15%)	> 0.99
Hemolytic anemia	0	1 (5%)	> 0.99
Covid + mother	2 (10%)	0	0.487
Anemia	0	1 (5%)	> 0.99
Mode of delivery			0.480
VD	7 (35%)	4 (20%)	
LSCS	13 (65%)	16 (80%)	
**Neonatal characteristics**			
Gestation (weeks) Median (IQR)	39.35 (36.33—40.075)	37.85 (35.40- 39.63)	0.254
Birth weight (grams) Median (IQR)	2792.50 (2465.00—3297.50)	2792.50 (2135.00- 3153.75)	0.286
Male gender	15 (75%)	14 (70%)	> 0.99
Weight for gestation age			0.762
SGA	4 (20%)	6 (30%)	
AGA	15 (75%)	13 (65%)	
LGA	1 (5%)	1 (5%)	
APGAR at 1 min Median (IQR)	5.00 (4.00- 7.00)	7.00 (6.00–8.00)	0.015
APGAR at 5 min Median (IQR)	7.00 (6.00- 8.00)	9.00 (8.00–9.00)	0.001
Sildenafil started at hours of life Median (IQR)	19.00 (10.25- 40.75)	30.00 (12.50- 35.00)	0.661
Initial pulmonary pressure (mm Hg) Median (IQR)	35.00 (32.00- 43.75)	39.00 (32.75- 58.25)	0.093
Underlying pathology			
MAS	10	6	0.102
Pneumoniae	4	9	0.091
Birth asphyxia	6	6	> 0.999
Sepsis	1	3	0.292
TTNB	1	0	0.311
RDS	0	1	0.311
Idiopathic	3	0	0.072
OI at starting sildenafil Median [IQR]	3.80 (2.20- 3.80) N = 15	3.70 (2.50- 8.30) N = 19	0.302
Full feeds at time of starting sildenafil	7 (35%) *N* = 20	2 (10%) *N* = 20	0.127

*AGA* appropriate for gestation age, *LGA* large for gestation age, *LSCS* lower segment caesarean section, *mm HG* millimetre of mercury, *MAS* meconium-stained amniotic fluid, *OI* oxygenation index, *IQR* interquartile range, *SGA* small for gestation age, *VD* vaginal delivery

At the onset of treatment, PAP was higher in IV sildenafil group, but was statistically insignificant (*p* = 0.093). Underlying pathologies in both the groups were similar. Sildenafil could be tapered in 17 (85%) and 15 (75%) neonates of the oral and IV groups, respectively. 15% of the neonates in the oral group and 25% in the IV group had to be started on other pulmonary vasodilators. Either iNO, milrinone or bosentan were used as rescue pulmonary vasodilator – on the discretion of treating physician. Sildenafil could be tapered after a median of 48 (IQR 24—96) hours in the oral group, compared to median of 48 (IQR 36 – 60) hours in the IV group (Table 2). After tapering Sildenafil, 3 neonates had rebound increase in PAP in the oral group, compared to 5 neonates in the IV group. Overall failure, defined as the need for iNO or any other pulmonary vasodilator in babies who are already on Sildenafil, or failure of reduction of PAP by 25% over 48 h was seen in 8 babies in the oral group and 9 babies in the IV group. In IV group—four babies developed hypotension and 1 developed poor cardiac contractility as assessed by echocardiography, compared to none in the oral group. All 5 required IV epinephrine infusion for the hemodynamic instability, which were required for 24–36 h. 18 babies in oral group and 19 babies in IV sildenafil group were discharged at median 11.5 (IQR 8.75–21.50) days and 19 (IQR 12–25) days respectively. Rest three had left against medical advice. All the outcome parameters were statistically insignificant except the complication rates, which were significantly more in the IV group (Table 2).

**Table 2:** Primary and Secondary outcomes

	Oral sildenafil	IV sildenafil	*P* value
Time (Hours) taken for pulmonary pressures to reduce below 25 mm Hg Median [IQR]	48 (24—96) (*N* = 17)	48 (36 – 60) (*N* = 15)	0.302
Time for OI to decrease by 25% (hours) Median [IQR]	12 (9–24) *N* = 9	24 (6–37.50) *N* = 14	0.281
Days on invasive ventilation Median [IQR]	4 (2–7.5) *N* = 8^a^	2.5 (2–3.75) *N* = 17^b^	0.963
Days on non-invasive ventilation Median [IQR]	5 (2–6.75) *N* = 20	6 (4.25–8.75) *N* = 20	0.831
Days on nasal oxygen Median [IQR]	2.50 (0–6) *N* = 13	0 (0–3) *N* = 9	0.530
Duration of hospital stay (Days) Median [IQR]	11.5 (8.75–21.50) *N* = 18	19 (12–25) *N* = 19	0.151
Outcome			> 0.99
Mortality	0	0	
Discharge	18	19	
Failure of treatment	8	9	> 0.99
Complications			0.049
Hypotension	0	4	
Poor cardiac contractility	0	1	
Reached full feeds on (DOL) Median [IQR]	5 (4–6.50) *N* = 13	4 (3–6.25) *N* = 18	0.361

*DOL* day of life, *OI* oxygenation index, *IQR* interquartile range

^a^Three out of 8 neonates in oral group required HFOV ventilation

^b^Six out of 17 neonates in IV group required HFOV ventilation

To check for the intra-observer and interobserver agreement, 20 echos were repeated at baseline, 12, 24, 36, 48, 60 and 72 h by the same observer and another clinician trained in functional echocardiography. There was good inter- and intra-observer agreement in all the pulmonary pressures measured with all interclass correlation coefficients (ICC) > 0.65.

## Discussion

Sildenafil, approved for use in adults for pulmonary hypertension, was first used in neonates with PPHN in 2002 [14]. The vasodilatation is mediated through inhibition of phosphodiesterase-5 (PDE5)-mediated breakdown of cyclic guanosyl monophosphate, resulting in its increased intracellular concentration, leading to smooth muscle relaxation [15].

Studies have documented the pulmonary vasodilatory role of sildenafil [3–6]. With limited availability of iNO or ECMO in resource limited NICUs, sildenafil has emerged as a drug of choice in neonates with pulmonary hypertension.

Studies have compared oral sildenafil with intravenous magnesium sulphate [10]. Also dose escalation study of intravenous sildenafil have been done [16]. There are no studies comparing the oral and intravenous route of administration of sildenafil for pulmonary hypertension in neonates. The comparison of the two routes of administration is important in view of the cost difference of the drugs, ease of oral administration, challenges of intravenous access and its maintainence, and conditions where enteral drug administration is not possible.

In this study, the time taken for the reduction of PAP was 48 h, similar to 2^nd^ day as seen in study by Uslu et al. [10]. The OI reduction in 12 h in the oral group and 24 h in the intravenous group, was comparable to 24 h in RCT done by Baquero et al. in 2006 [4]. A 2009 dose-escalation open trial by Steinhorn et al. showed that IV sildenafil was well tolerated and led to a significant improvement in OI (28.7 to 19.3) after 4 h of infusion [16]. In our study, since the parameters were monitored echocardiographically twelve hourly, the study design could not have detected this difference.

Duration of invasive mechanical ventilation was 4 days in oral group and 2.5 days in IV sildenafil group in our study. This was similar to study by Uslu et al., [10] where ventilation was required for 4 days and the study by Vargas-Origel et al., [5] where ventilation was required for 5 days in neonates with pulmonary hypertension on oral sildenafil.

There was no mortality in the study cohort during hospital stay. This is in contrast with mortality rates of 5–15% in other studies assessing the response of sildenafil in cases of pulmonary hypertension [4, 5, 8]. This may be due to difference in studied population, as in our study mild to moderate cases of pulmonary hypertension were recruited as against severe cases of pulmonary hypertension in other studies.

Hypotension was observed in four neonates receiving intravenous sildenafil in this study. Similar event has been reported in the dose-escalation open trial by Steinhorn et al. [16]. Poor cardiac contractility was observed in one neonate on IV sildenafil in our study. Though this has not been reported in other studies. None of the neonates receiving oral sildenafil had these events during the study period, similar to other studies [4, 10, 17]. In our study we compared the efficacy of oral and IV sildenafil in babies with pulmonary hypertension due to any cause diagnosed before 72 h of life. It was found that neonates in the oral sildenafil group, despite having worse APGAR scores at birth, showed response that was similar to the intravenous sildenafil group in terms of time taken for PAP to reduce to 25 mm Hg, requirement of any other pulmonary vasodilator, rebound of pulmonary hypertension after tapering dose of sildenafil, time to reach full feeds, duration of non-invasive and invasive respiratory support and duration of hospital stay. However, the complication rate in the IV sildenafil group was higher, with hypotension (4/20) and poor cardiac contractility (1/20) compared to none in oral sildenafil group.

The main limitation of the present study is that very sick babies requiring iNO at the beginning were excluded from the study, so the findings are not applicable for severe pulmonary hypertension. Some cases of pulmonary hypertension could have been missed as pulmonary pressures were measured only by tricuspid regurgitation. Also, resolution of high PAP was due to treatment of aetiology per se or due to sildenafil cannot be ascertained, though the pulmonary vasodilator was added for persistent pulmonary hypertension only after ensuring adequate ventilation and other supportive measures like surfactant, antibiotics. Being the pilot study, convenient sample size was taken based on the admission rates and the morbidity profile of the cases admitted in the unit in the preceding years. Blinding could not be done as the treating clinicians and investigators were the same. Also, the cut off of 25 mm Hg as inclusion criteria and the response to sildenafil as time taken for the pressures to reduce below 25 mm Hg may overlap with the normal physiology. Though, these neonates being symptomatic with requirement of respiratory support – may indicate the pathological pulmonary pressures.

But with in-house availability of the echocardiography machine, echocardiographic assessments could be done every 12 h, which increased the sensitivity of detecting change in pulmonary hypertension. Also, the trial focused on the problem of most of the resource limited NICUs where many neonates present with high Fio2 requirement disproportionate to the lung parenchymal disease. This trial focuses on those neonates with mild to moderate pulmonary hypertension with any cause, where sildenafil is used.

Pharmacokinetic studies to compare the serum level of sildenafil and its metabolite after administration of IV and oral drugs using ultra-performance liquid chromatography with tandem mass spectrometry detection in combination with pharmacokinetic modelling software can help compare the routes of administration using limited number and amount of plasma samples [18].

## Conclusion

Role of sildenafil in neonatal pulmonary hypertension in resource poor settings is already proven. This study highlights the benefit of both routes of administration of sildenafil for pulmonary hypertension in neonates, but cautions for close monitoring for hypotension and cardiac contractility, while using intravenous route for sildenafil administration. Further multicentric blind studies with larger sample size are needed for comparing the two routes of administration of sildenafil preparation for pulmonary hypertension in neonates for the efficacy and safety profile.

## Supplementary Information

**Additional file 1.** 

## Data Availability

The datasets used and/or analyzed during the current study are being provided as supplementary file.

## Abbreviations

ECMO: Extracorporeal Membrane Oxygenation
ICC: Interclass Correlation Coefficients
iNO: Inhaled Nitric Oxide
IQR: Interquartile Range
IV: Intravenous
NEC: Necrotizing Enterocolitis
NICU: Neonatal Intensive Care Unit
OI: Oxygenation Index
PAP: Pulmonary Arterial Pressure
PDE5: Phosphodiesterase-5
PPHN: Persistent Pulmonary Hypertension of Newborn
RCT: Randomized Control Trial
